# Time in LDL cholesterol target range and major adverse cardiovascular events risk: A pooled analysis of two cohorts

**DOI:** 10.1016/j.ajpc.2025.101290

**Published:** 2025-09-10

**Authors:** Yezhou Liu, Zhe Lv, Hong Yan, Yamei Liu, Jiaheng Zhang, Wenming Bian, Yetong Liu, Zhaojie Song, Peng Han, Tao Chen, Chao Li

**Affiliations:** a Department of Epidemiology and Health Statistics, Xi’an Jiaotong University Health Science Center, Xi’an, Shaanxi, China; b Xi’an Children’s Hospital, Xi’an, Shaanxi, China; c Department of Otolaryngology-Head and Neck Surgery, the First Affiliated Hospital of Xi'an Jiaotong University, Xi'an, Shaanxi, China; d Department of Clinical Sciences, Liverpool School of Tropical Medicine, Liverpool, UK; e Key Laboratory of Environment and Genes Related to Diseases (Xi'an Jiaotong University), Ministry of Education, Xi'an, Shaanxi, China

**Keywords:** Low-density lipoprotein, Time in target range, Major adverse cardiovascular events, Myocardial infarction, Stroke, Cardiovascular death

## Abstract

**Background:**

Traditional management of low-density lipoprotein cholesterol (LDL-C) relies on single-point measurements, neglecting long-term magnitude and the duration of exposure to elevated LDL-C over time. This study aimed to evaluate the association between LDL-C time in target range (TTR) and the risk of major adverse cardiovascular events (MACE) in two US cohorts.

**Methods:**

This study was a secondary analysis of the Atherosclerosis Risk in Communities (ARIC) study and the Multi-Ethnic Study of Atherosclerosis (MESA) cohorts. LDL-C TTR was defined as the percentage of the area under curve for LDL-C values below 100 mg/dL relative to the total area over the first 4 visits. Association between LDL-C TTR and MACE was estimated using adjusted Cox models and Fine-Gray competing risk models.

**Results:**

Over a mean follow-up of 21.2 ± 8.1 years, 3306 MACE occurred among 16,310 participants. The highest LDL-C TTR quartile was associated with a 25 % reduction in MACE (HR: 0.75, 95 % CI: 0.68 to 0.83), 42 % reduction in myocardial infarction (HR: 0.58, 95 % CI: 0.49 to 0.68), 24 % reduction in stroke (HR: 0.76, 95 % CI: 0.64 to 0.91), and 13 % reduction in CVD death (HR: 0.87, 95 % CI: 0.76 to 1.00; borderline significance). TTR remained significantly associated with MACE after adjusting for mean LDL-C or LDL-C variability, and were robust in competing risk analyses. TTR also outperformed mean LDL-C and LDL-C variability in prognostic value (Akaike information criterion, C-statistics).

**Conclusions:**

LDL-C TTR independently predicts MACE and may offer a more comprehensive assessment of long-term LDL-C control than mean levels or variability, supporting its potential clinical utility.

## Graphical abstract

**Figure:**
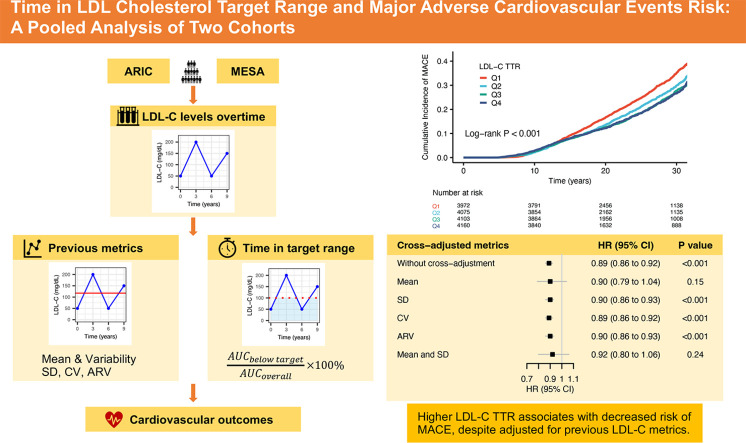

## 1 Background

Effective management of low-density lipoprotein cholesterol (LDL-C), a critical target in cholesterol control, has consistently been shown to reduce the risk of cardiovascular disease (CVD) events [[1], [2], [3]]. Current US cholesterol guideline recommend maintaining an optimal LDL-C level below 100 mg/dL (approximately 2.6 mmol/L), with even stricter targets for individuals at high risk for CVD [4].

Despite its clinical significance, LDL-C is a continuous and dynamic variable. In practice, monitoring often relies on a single or average LDL-C value, which may inadequately capture its true variability [4,5]. Recent studies have demonstrated that cumulative exposure to elevated LDL-C is associated with an earlier onset of major adverse cardiovascular events (MACE) [[6], [7], [8]]. Additionally, emerging evidence suggests that greater fluctuations in LDL-C, measured through visit-to-visit variability metrics such as standard deviation (SD), coefficient of variation (CV), or average real variability (ARV), are linked to an increased risk of cardiovascular outcomes [[9], [10], [11], [12]]. These findings underscore the need for continuous LDL-C monitoring, rather than relying solely on measurements taken at a single time point.

In prior post hoc analyses of two trials, we assessed the impact of time in target range (TTR) for systolic blood pressure (BP) (120 to 130 mm Hg) on the risk of cardiovascular death or heart failure hospitalization [13]. This approach accounts for both long-term average values and variability over time. However, the original concept of TTR fails to capture the magnitude of change over time. To address this limitation, we proposed a revised TTR metric, calculating the percentage of the area under the curve (AUC) relative to the AUC for all measured LDL-C values. Our primary objective is to assess the association between LDL-C TTR and the risk of MACE in two cohorts. Furthermore, we compared its predictive performance with mean LDL-C levels and visit-to-visit variability.

## 2 Methods

### 2.1 Data source and study population

This study is a post hoc pooled analysis using limited-access datasets from two large US cohort studies: the ARIC (the Atherosclerosis Risk in Communities) study (NCT00005131) and the MESA (Multi-Ethnic Study of Atherosclerosis, NCT00005487). Data were obtained from the National Heart, Lung, and Blood Institute’s Biologic Specimen and Data Repository Information Coordinating Center (NHLBI BioLINCC) via an approved proposal.

The rationale and design of ARIC and MESA have been published previously [14,15]. The ARIC study, initiated in 1987, recruiting White and Black adults aged 45 to 64 years from four US communities. Participants were followed up annually (semiannually since 2012) by telephone to monitor health status. In-person visits were conducted to collect physical examination data and biological samples, with the first four visits conducted approximately every three years. Additionally, mortality status was ascertained through linkage to the National Death Index (NDI). For this analysis, we included data up until the end of 2019, which covered seven in-person visits and approximately 30 years of follow-up.

The MESA study began in 2000, enrolling adults aged 45 to 84 who were free of clinical CVD from four race/ethnic groups across six field centers in the United States. Participants were contacted by telephone every 6 to 12 months to track their health, and in-person examinations were conducted approximately every two years during the first four visits. CVD events during follow-up were classified using various data sources, including public files (death certificates), medical records from hospitalizations, autopsy reports, and participant interviews. For this analysis, we used data available up to the end of 2015, covering five in-person visits and approximately 15 years of follow-up.

In this study, we included participants with at least three LDL-C measurements from the first four visits. Individuals with coronary heart diseases (CHD) or stroke at baseline, as well as those who experienced cardiovascular outcomes during the first four visits, were excluded. The final sample comprised 16,310 individuals. Demographic characteristics, LDL-C levels, and other risk factors were measured in each cohort according to standardized protocols in both cohorts and were harmonized and pooled for analysis. Both cohort studies received approval from the institutional review boards at their respective participating institutions. Since this study involved secondary data analysis, it was granted a waiver of ethical approval by the Ethical Review Board of the First Affiliated Hospital of Xi’an Jiaotong University.

### 2.2 LDL-C measurements and definition of TTR

In both ARIC and MESA, fasting blood samples were collected and analyzed in central laboratories following protocols standardized according to the Centers for Disease Control and Prevention (CDC) and NHLBI. In ARIC, total cholesterol (TC), HDL-C, and triglycerides (TG) were measured enzymatically using a Cobas-Bio Analyzer© (Roche) [16]. In MESA, TC, HDL-C and TG was measured using standardized Roche assays [17]. In both cohorts, LDL-C was calculated using the Friedewald equation for participants with TG <400 mg/dL [18], while those with TG ≥400 mg/dL did not have calculated LDL-C values and were excluded.

TTR for the first 4 visits was calculated as the percentage of the AUC for LDL-C values below 100 mg/dL, relative to the AUC for all measured LDL-C values (see **Supplementary Fig. 1**). The AUC was calculated using the trapezoid rule, connecting all data points while assuming a linear relationship between two consecutive LDL-C measurements. The target range was defined as LDL-C <100 mg/dL (2.6 mmol/L), in alignment with current cholesterol guidelines for normal LDL-C levels [4].

Mean LDL-C during the initial 4 visits was calculated as the average LDL-C value across all measurements. Visit-to-Visit variability was assessed using the three indices: standard deviation (SD), coefficient of variation (CV), and average real variability (ARV). ARV was calculated as the mean absolute difference between successive LDL-C measurements.

### 2.3 Study outcomes

The primary outcome was major adverse cardiovascular events (MACE), defined as the first occurrence of either myocardial infarction (MI), stroke, or cardiovascular disease (CVD) death. In ARIC, MI were defined as adjudicated definite and probable MI, stroke as adjudicated definite or probable stroke, and CVD death according to ICD codes: 390–459 for ICD-9 and I00–I99 ICD-10. Participants in ARIC were followed until either administrative censoring on December 31, 2019, the occurrence of the specified outcome, or loss to follow-up, whichever came first. In MESA, MI and stroke were similarly adjudicated, and CVD death was categorized as atherosclerotic CHD death, stroke death, other atherosclerotic disease death (non-coronary/non-stroke), or other CVD death. Participants in MESA were followed until December 31, 2015, the occurrence of the specific outcome, or loss to follow-up, whichever came first.

### 2.4 Statistical analysis

Baseline characteristics of participants were summarized for the pooled cohort, stratified by quartiles of LDL-C TTR and by each cohort. Data were presented as counts and percentages or mean ± SD, as appropriate. Correlations between LDL-C TTR and other indices (mean LDL-C, LDL-C SD, LDL-C CV, and LDL-C ARV) were explored using scatter plots and Pearson correlation coefficients (Pearson *r*).

Cox proportional hazards regression models, stratified by cohort, were used to evaluate the association between the five LDL-C indices and each CVD outcome with covariables added sequentially. The proportional hazard assumption was tested using Schoenfield residual plots, and all five LDL-C indices met the assumption (**Supplementary Fig. 3**). Hazard ratios (HRs) and 95 % confidence intervals (CIs) were reported. A linear trend across quartiles of LDL-C TTR was tested by treating the quartiles as a continuous variable (ranging from 1 to 4). The log-rank test was applied for univariate association, and Kaplan-Meier plots were generated for visualization. HRs per 1-SD increase were also reported and compared with other indices. The main analysis was repeated for each cohort individually.

To account for competing risks, Fine-Gray subdistribution hazard models were applied. For MACE and CVD death, non-CVD death was treated as a competing event; for MI and stroke, all-cause death was considered a competing event. Models accounted for cohort-specific censoring by specifying cohort as a grouping variable (cengroup) and were adjusted for the same set of covariates as in Cox model 3. Subdistribution hazard ratios (sHRs) and 95 % confidence intervals (CIs) were reported.

We additionally assessed the independent association of LDL-C TTR with CVD outcomes by adding mean LDL-C, LDL-C SD, CV, ARV solely, and both mean LDL-C and SD together to the models alongside LDL-C TTR. A forest plot of HRs and 95 % CIs for the primary outcome was also generated. Moreover, we repeated the analysis for LDL-C TTR stratified by mean LDL-C quartile groups.

To evaluate the predictive values of each LDL-C index in addition to a base model of traditional risk factors for the primary outcome, we compared C-statistics. C-statistics and differences were calculated with 95 % bootstrap CIs based on percentiles of the empirical bootstrap distribution with 1000 iterations. The Akaike information criterion (AIC) was used to compare models and determine the best-fitting models with the lowest AIC indicating the best overall fit.

All analyses were conducted using R version 4.3.1 (R Foundation for Statistical Computing).

## 3 Results

### 3.1 Basic characteristics

**Fig. 1:**
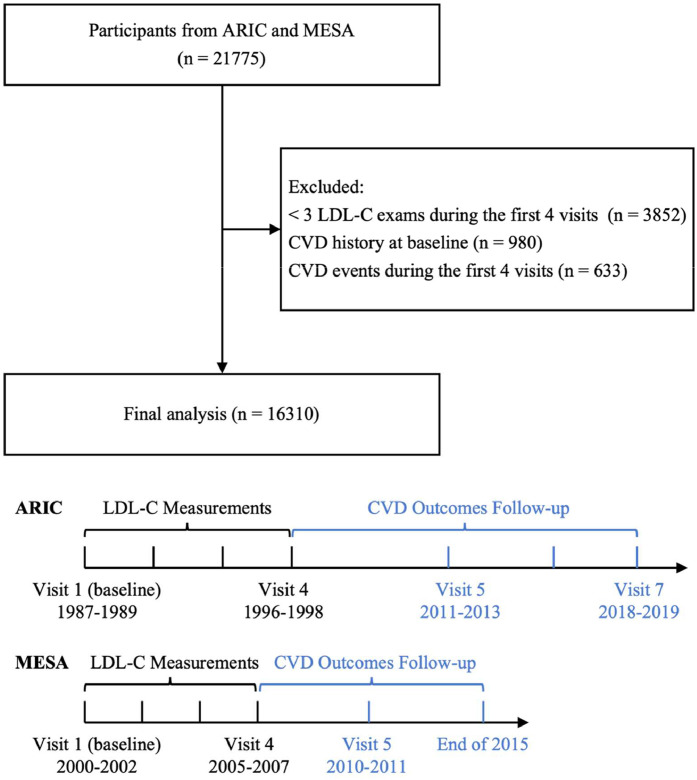
Flowchart of participants selection and study timeline.

Among the 21,775 participants initially available from the ARIC and MESA cohorts, we excluded those with fewer than three LDL-C measurements during the first four visits (*n* = 3852), individuals with a history of CVD at baseline (*n* = 980), and those who experienced CVD events during the first 4 visits (*n* = 633). This resulted in a final analytical sample of 16,310 individuals (Fig. 1), with 66 % from the ARIC cohort (*n* = 10,835) and 34 % from the MESA cohort (*n* = 5475).

The mean age of the pooled population was 56.4 ± 8.2 years, with 9127 (56.0 %) women. Most participants had four LDL-C measurements during the first four visits (*n* = 14,071, 86.3 %), spanning a duration of 7.3 ± 2.0 years, with a mean interval of 2.6 ± 0.7 years between measurements (**Supplementary Table 1**). The overall LDL-C TTR across the first 4 visits was 80.6 ± 14.3 %, and the mean LDL-C level during these visits was 124.4 ± 30.5 mg/dL. The overall LDL-C SD, CV and ARV were 18.0 ± 11.6 mg/dL, 0.1 ± 0.1, and 20.5 ± 13.8 mg/dL, respectively (Table 1**, Supplementary Table 1**). Characteristics of participants from the ARIC and MESA cohorts are also detailed in **Supplementary Table 1**.

**Table 1:** Characteristics of participants by LDL-C TTR quartiles.

		LDL-C TTR			
	Overall	29.6 % to <69.2 %	69.2 % to <80.6 %	80.6 % to <93.5 %	93.5 % to 100.0 %
Characteristic	*n* = 16,310	*n* = 3972	*n* = 4075	*n* = 4103	*n* = 4160
Cohorts					
ARIC	10,835 (66.4)	3339 (84.1)	2859 (70.2)	2492 (60.7)	2145 (51.6)
MESA	5475 (33.6)	633 (15.9)	1216 (29.8)	1611 (39.3)	2015 (48.4)
Gender					
Female	9127 (56.0)	2284 (57.5)	2254 (55.3)	2318 (56.5)	2271 (54.6)
Male	7183 (44.0)	1688 (42.5)	1821 (44.7)	1785 (43.5)	1889 (45.4)
Age, years	56.4 ± 8.2	55.2 ± 6.6	55.9 ± 7.6	56.8 ± 8.7	57.8 ± 9.5
White	10,701 (65.6)	2770 (69.7)	2844 (69.8)	2635 (64.2)	2452 (58.9)
Educational level					
Below high school	3889 (23.9)	923 (23.3)	922 (22.7)	977 (23.8)	1067 (25.7)
High school graduate or GED	5853 (36.0)	1652 (41.7)	1484 (36.5)	1436 (35.1)	1281 (30.9)
College or above	6538 (40.2)	1390 (35.1)	1661 (40.8)	1684 (41.1)	1803 (43.4)
Smoking					
Never	7666 (47.1)	1860 (46.9)	1937 (47.6)	1917 (46.8)	1952 (47.0)
Former	5564 (34.2)	1287 (32.5)	1368 (33.6)	1445 (35.2)	1464 (35.2)
Current	3059 (18.8)	818 (20.6)	765 (18.8)	738 (18.0)	738 (17.8)
Drinking					
Never	3734 (23.0)	1006 (25.4)	891 (21.9)	901 (22.1)	936 (22.6)
Former	3025 (18.6)	744 (18.8)	737 (18.1)	749 (18.3)	795 (19.2)
Current	9479 (58.4)	2204 (55.7)	2434 (59.9)	2434 (59.6)	2407 (58.2)
BMI, kg/m^2^	27.8 ± 5.3	27.9 ± 4.9	27.8 ± 5.2	27.7 ± 5.3	27.6 ± 5.6
MVPA, MET-min/wk	955.6 ± 1603.3	760.6 ± 1328.0	928.0 ± 1495.0	1030.2 ± 1795.6	1095.4 ± 1722.2
Cholesterol-lowering Medication Use	1139 (7.0)	193 (4.9)	177 (4.4)	285 (7.0)	484 (11.7)
Hypertension	5578 (34.3)	1245 (31.4)	1315 (32.4)	1414 (34.6)	1604 (38.6)
Diabetes	1533 (9.4)	333 (8.4)	348 (8.6)	378 (9.3)	474 (11.4)
Mean LDL-C, mg/dL	124.4 ± 30.5	164.0 ± 19.1	133.2 ± 7.2	114.0 ± 7.2	88.2 ± 14.1
LDL-C SD, mg/dL	18.0 ± 11.6	22.5 ± 14.5	18.9 ± 11.4	17.7 ± 10.2	13.2 ± 7.3
LDL-C CV	0.1 ± 0.1	0.1 ± 0.1	0.1 ± 0.1	0.2 ± 0.1	0.2 ± 0.1
LDL-C ARV, mg/dL	20.5 ± 13.8	25.6 ± 17.4	21.5 ± 13.6	19.8 ± 12.0	15.3 ± 9.2

Data are shown as n (%) or Mean ± SD.

There were 30 missing values for educational level, 21 for smoking, 72 for drinking, 8 for BMI, 18 for MVPA, 81 for cholesterol-lowering medication use, 44 for hypertension, and 65 for diabetes.

Abbreviations:.

ARIC: Atherosclerosis Risk in Communities study; MESA: Multi-Ethnic Study of Atherosclerosis; GED, general educational development; BMI: body mass index; MVPA: moderate and vigorous physical activity; LDL-C: low density lipoprotein cholesterol; SD: Standard deviation; CV: coefficient of variation; ARV, average real variability; TTR: time in target range.

SI conversion factor: To convert cholesterol levels to millimoles per liter, multiply by 0.0259.

Participants with higher LDL-C TTR were more likely to be from the MESA cohort, younger, male, Non-White, with higher education, former smokers, more physically active, users of cholesterol-lowering medication, and with hypertension and diabetes. Drinking status and BMI were similar across LDL-C TTR quartiles (Table 1).

**Fig. 2:**
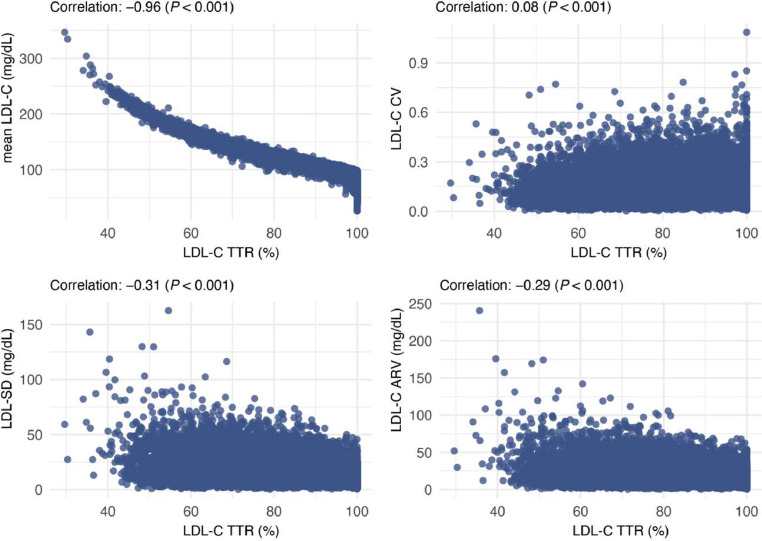
Scatterplots of LDL-C TTR and other LDL-C Indices.

LDL-C TTR was strongly reversely correlated with mean LDL-C (*r* = −0.96, *P* <0.001) but showed weaker correlations with LDL-C variability indices (*r* = −0.31, *P* <0.001 for SD; *r* = 0.08, *P* <0.001 for CV; *r* = −0.29, *P* <0.001 for ARV) (Fig. 2).

### 3.2 Association of LDL-C TTR with CVD outcomes

Over a mean follow-up period of 21.2 ± 8.1 years, there were 3306 MACE, 1288 MI, 1090 stroke and 1694 CVD deaths. As shown in **Supplementary Fig. 4**, cumulative incidences of MACE significantly decreased across LDL-C TTR quartiles, with the lowest incidence in the top quartile (*P* <0.0001). Similar trends were observed for MI, stroke, and CVD mortality, although the association with stroke was not statistically significant.

Further analysis, adjusted multiple confounders, consistently showed that the highest LDL-C TTR quartile was associated with the lowest HR for all CVD outcomes (Table 2). In the fully adjusted model, the highest LDL-C TTR quartile was associated with a 25 % reduction in MACE risk (HR: 0.75, 95 % CI: 0.68 to 0.83), 42 % reduction in MI risk (HR: 0.58, 95 % CI: 0.49 to 0.68), 24 % reduction in stroke risk (HR: 0.76, 95 % CI: 0.64 to 0.91), and 13 % reduction in CVD death risk (HR: 0.87, 95 % CI: 0.76 to 1.00), the latter reflecting borderline significance. Additional analysis by cohort confirmed the association between LDL-C TTR with MACE, especially in the ARIC cohort population, where the highest quartile of LDL-TTR was associated with a 22 % reduction in the MACE (HR: 0.78, 95 % CI: 0.70 to 0.87) (**Supplementary Table 2**). During follow-up, competing events often outnumbered the target outcomes (e.g., for MACE: 3256 events vs. 3508 competing events; Supplementary Table 2a).Results were consistent in competing risk analyses using Fine-Gray models. For example, the highest LDL-C TTR quartile was associated with a 29 % lower subdistribution hazard of MACE (sHR: 0.71, 95 % CI: 0.64 to 0.78). Similar associations were observed for MI, stroke, and CVD death (Supplementary Table 2a).

**Table 2:** Associations of LDL-C TTR and cardiovascular outcomes, HR (95 %CI).

				Quartiles of LDL-C TTR				
		N	N of Event	Q1	Q2	Q3	Q4	*P* for trend
				29.6 % to <69.2 %	69.2 % to <80.6 %	80.6 % to <93.5 %	93.5 % to 100.0 %	
MACE	Unadjusted	16,280	3306	1 (ref.)	0.81 (0.74 to 0.89)	0.73 (0.66 to 0.80)	0.73 (0.66 to 0.80)	<0.001
	Model 1	16,280	3306	1 (ref.)	0.85 (0.78 to 0.94)	0.79 (0.71 to 0.87)	0.79 (0.71 to 0.87)	<0.001
	Model 2	16,204	3291	1 (ref.)	0.86 (0.78 to 0.94)	0.79 (0.72 to 0.87)	0.79 (0.72 to 0.88)	<0.001
	Model 3	16,046	3256	1 (ref.)	0.83 (0.76 to 0.91)	0.78 (0.71 to 0.86)	0.75 (0.68 to 0.83)	<0.001
								
MI	Unadjusted	16,280	1288	1 (ref.)	0.77 (0.67 to 0.89)	0.56 (0.48 to 0.65)	0.57 (0.49 to 0.67)	<0.001
	Model 1	16,280	1288	1 (ref.)	0.79 (0.69 to 0.91)	0.58 (0.50 to 0.68)	0.60 (0.51 to 0.71)	<0.001
	Model 2	16,204	1280	1 (ref.)	0.80 (0.69 to 0.92)	0.59 (0.51 to 0.70)	0.61 (0.52 to 0.72)	<0.001
	Model 3	16,046	1265	1 (ref.)	0.78 (0.68 to 0.90)	0.58 (0.50 to 0.68)	0.58 (0.49 to 0.68)	<0.001
								
Stroke	Unadjusted	16,280	1090	1 (ref.)	0.83 (0.71 to 0.98)	0.85 (0.72 to 1.00)	0.75 (0.63 to 0.89)	0.002
	Model 1	16,280	1090	1 (ref.)	0.89 (0.75 to 1.04)	0.92 (0.78 to 1.08)	0.81 (0.68 to 0.96)	0.033
	Model 2	16,204	1085	1 (ref.)	0.89 (0.76 to 1.04)	0.93 (0.79 to 1.09)	0.81 (0.68 to 0.96)	0.035
	Model 3	16,046	1075	1 (ref.)	0.87 (0.74 to 1.02)	0.91 (0.78 to 1.08)	0.76 (0.64 to 0.91)	0.009
								
CVD Death	Unadjusted	16,280	1694	1 (ref.)	0.79 (0.70 to 0.90)	0.76 (0.67 to 0.87)	0.80 (0.70 to 0.92)	<0.001
	Model 1	16,280	1694	1 (ref.)	0.85 (0.75 to 0.96)	0.85 (0.75 to 0.97)	0.91 (0.79 to 1.05)	0.12
	Model 2	16,204	1689	1 (ref.)	0.85 (0.75 to 0.96)	0.85 (0.75 to 0.98)	0.93 (0.81 to 1.06)	0.17
	Model 3	16,046	1670	1 (ref.)	0.82 (0.72 to 0.93)	0.84 (0.74 to 0.96)	0.87 (0.76 to 1.00)	0.04

Abbreviations:.

HR: hazard ratio; CI: confidence interval; MACE: major adverse cardiovascular event; CVD: cardiovascular disease; MI: myocardial infarction; LDL-C, low density lipoprotein cholesterol; TTR, time in target range.

MACE was defined as the first occurrence of MI, stroke, and cardiovascular death.

Cox models with cohort strata were deployed.

Model 1: adjusted for age, gender, race, and education level;.

Model 2: adjusted for model 1 + smoking status, drinking status, body mass index, and moderate-vigorous physical activity;.

Model 3: adjusted for model 2 + use of cholesterol-lowering medicines, prevalent hypertension, and prevalent diabetes.

### 3.3 Independent association of LDL-C TTR, mean LDL-C and LDL-C variability with CVD outcomes

**Fig. 3:**
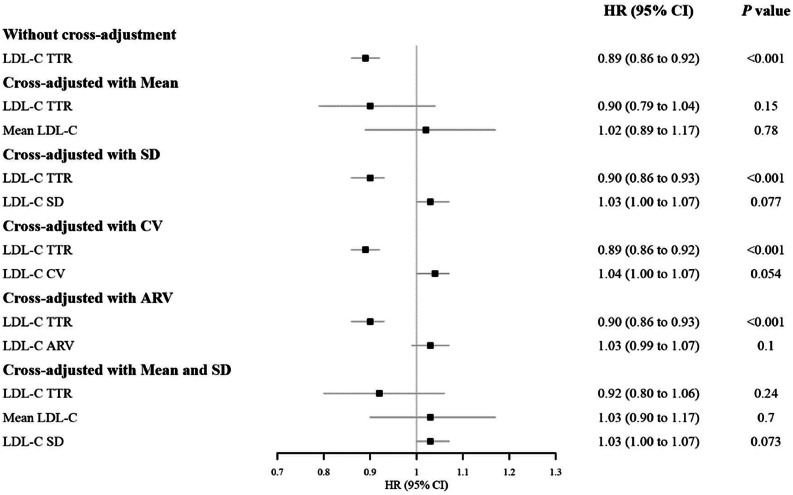
Cross-adjusted association of LDL-C TTR and LDL-C indices and MACE. HR per 1-SD increase in LDL-C TTR, mean LDL-C, LDL-C SD, LDL-C CV, and LDL-C ARV. Abbreviations: LDL-C: low density lipoprotein cholesterol; SD: Standard deviation; CV: coefficient of variation; ARV, average real variability; TTR: time in target range. Cox models with cohort strata were deployed. Adjusted for age, gender, race, education level, smoking status, drinking status, body mass index, moderate-vigorous physical activity, use of cholesterol-lowering medicines, prevalent hypertension, and prevalent diabetes.

Each 1-SD increase in LDL-C TTR consistently indicated a significantly reverse association with MACE risk across different analysis models (**Supplementary Tables 3 and 4**), even after cross-adjusting for different LDL-C variability indices, though this association lost significance while adjusting mean LDL-C (Fig. 3**)**. Additional stratified analyses by mean LDL-C quartiles suggested that a higher LDL-C TTR was significantly associated with a decreased risk of MACE in the highest quartile (HR: 0.92, 95 % CI: 0.87 to 0.96) (Table 3). Similar patterns were persistent for CVD death, MI, and stroke. Conversely, mean LDL-C, SD, CV, and ARV were not significantly associated with certain secondary outcomes. For example, 1.00 (0.94 to 1.06) with stroke for SD, 1.03 (0.83 to 1.28) with MI for mean LDL-C after cross-adjusting for LDL-C TTR (**Supplementary Tables 3 and 4)**.

**Table 3:** Fully adjusted associations of LDL-C TTR and cardiovascular outcomes stratified by mean LDL-C quartile groups.

	Q1		Q2		Q3		Q4	
	25.75 to <103.65 mg/dL		103.65 to <123 mg/dL		123 to <143.5 mg/dL		143.5 to 346.5 mg/dL	
Outcome	HR (95 % CI)	*P* value	HR (95 % CI)	*P* value	HR (95 % CI)	*P* value	HR (95 % CI)	*P* value
MACE	0.96 (0.89 to 1.04)	0.36	0.98 (0.91 to 1.05)	0.53	0.98 (0.91 to 1.05)	0.5	0.92 (0.87 to 0.98)	0.006
MI	0.99 (0.86 to 1.13)	0.84	0.91 (0.80 to 1.04)	0.17	0.90 (0.81 to 1.00)	0.061	0.86 (0.79 to 0.94)	<0.001
Stroke	0.94 (0.83 to 1.07)	0.34	0.97 (0.86 to 1.10)	0.69	0.99 (0.88 to 1.12)	0.93	0.98 (0.88 to 1.09)	0.73
CVD Death	0.96 (0.86 to 1.07)	0.42	1.03 (0.93 to 1.14)	0.56	0.98 (0.89 to 1.08)	0.65	0.96 (0.89 to 1.04)	0.33

HR per 1-SD increase in LDL-C TTR.

Abbreviation:.

HR: hazard ratio; CI: confidence interval; MACE: major adverse cardiovascular event; MI: myocardial infarction; LDL-C: low density lipoprotein cholesterol; and TTR: time in target range.

MACE was defined as the first occurrence of MI, stroke, and cardiovascular death.

Cox models with cohort strata were deployed.

Adjusted for age, gender, race, education level, smoking status, drinking status, body mass index, moderate-vigorous physical activity use of lowering-cholesterol drugs, prevalent hypertension, and prevalent diabetes.

### 3.4 Prognostic value of LDL-C TTR compared with other LDL-C indices

For MACE, adding LDL-C TTR to the base model (only adjusted traditional risk factors) provided the best prognostic value compared with other LDL-C indices, as evidenced by the lowest AIC (AIC: 57,127.97) and the largest improvement of C-statistics (difference in C-statistics: 0.0026, 95 % CI: −0.0103 to 0.0155). (**Supplementary Table 5**).

## 4 Discussion

Our study demonstrates a significant inverse association between LDL-C TTR, a novel metric that captures both LDL-C levels and the time spent within target ranges, and MACE in two large and diverse prospective cohorts of US adults. Maintaining an LDL-C TTR above 70 % during midlife was associated with at least a 17 % reduction in the risk of MACE later in life. This association remained robust after adjusting for demographic, socioeconomic, lifestyle, and clinical factors, suggesting that LDL-C TTR is an independent predictor of CVD outcomes compared to other LDL-C indices. Importantly, these associations were consistent in competing risk analyses using Fine-Gray models, further reinforcing the robustness of our findings against potential bias from non-CVD deaths. These findings suggest that LDL-C TTR may serve as a more comprehensive measure of cholesterol management than mean LDL-C levels or visit-to-visit variability.

Given that LDL-C levels fluctuate over time, relying on a single measurement may fail to capture liquid-related cardiovascular risk. While numerous observational studies [[19], [20], [21]] and randomized trials [2,3,22,23] have established a dose-dependent association between absolute LDL-C levels and risk of cardiovascular events, maintaining stable LDL-C levels with minimal variability is also crucial, as greater visit-to-visit variability is an independent predictor of cardiovascular outcomes [[9], [10], [11], [12]]. However, a comprehensive measure integrating both LDL-C magnitude and variability of LDL-C has been lacking, as prior studies have primarily focused on these aspects individually.

In the field of hypertension management, the concept of “time in target range” (TTR) has been proposed as a more accurate measure that reflects both average levels and variability over time [13,[24], [25], [26], [27], [28], [29], [30]]. This concept, which offers an alternative to single time-point measurements, has also been applied to other continuous metrics such as blood glucose [[31], [32], [33]], body weight [34,35], physical activity [36], and resting heart rate [37]. However, it has not yet been explored for LDL-C management. Recent studies emphasize the importance of cumulative LDL-C exposure over long periods [[6], [7], [8],^38^], highlighting the benefit of achieving and maintaining LDL-C levels below target thresholds from an early age. Neither the original TTR concept nor cumulative exposure sufficiently distinguishes between individuals with transiently high LDL-C levels and those with moderately elevated LDL-C levels sustained over a longer duration.

Wang et.al applied the concept of cumulative BP load [39], which simultaneously accounts for both time and magnitude by calculating the area over a target BP threshold relative to the overall BP area over time. Their study found cumulative BP load to be an independent predictor of MACE. In our study, we adapted this concept by calculating LDL-C TTR as the AUC below the target threshold relative to the overall AUC, capturing both the duration and magnitude of LDL-C exposure. Our findings suggest that LDL-C TTR may provide additional value beyond currently used LDL-C control metrics and could be of major clinical importance for LDL-C monitoring. To the best of our knowledge, this is the first study to explore LDL-C TTR as a protective factor against MACE, demonstrating that maintaining LDL-C levels at or below 100mg/dL for extended periods is associated with a reduced risk of cardiovascular events. Furthermore, LDL-C TTR demonstrated better prognostic value than other LDL-C indices. This study suggests that sustained control of LDL-C within target ranges may be as important as reducing average LDL-C levels or minimizing fluctuations for preventing CVD.

In this study, the primary outcome was MACE, for which we observed a clear and graded inverse association with LDL-C TTR across quartiles. For secondary outcomes (MI, stroke, and CVD death), associations were directionally consistent, though precision varied. The association with MI were strong and stable across models. Stroke results were less precise due to fewer events, but both Cox models (*P* for trend = 0.009) and Fine-Gray competing risk analyses (*P* for trend = 0.005) supported a significant inverse trend, suggesting limited power rather than absence of an association. By contrast, CVD death showed only borderline significance in Cox models (Q4 vs. Q1, HR: 0.87, 95 % CI: 0.76 to 1.00), though robustness was supported in competing risk analyses (sHR: 0.77, 95 % CI: 0.67 to 0.88). Notably, in both cohorts, competing events (particularly non-CVD deaths) substantially outnumbered cardiovascular events (e.g., for MACE: 3256 vs. 3508 competing events; for stroke: 1075 vs. 5234 competing events; for CVD death, 1670 vs. 4222 Non-CVD death), underscoring the importance of explicitly accounting for them. Applying Fine-Gray subdistribution hazard models confirmed that the protective associations of LDL-C TTR remained consistent across all outcomes, reinforcing the robustness of our findings. Nevertheless, some heterogeneity in effect sizes was observed between ARIC and MESA, which is important to consider in interpreting our results.

We also observed heterogeneity by cohort. Associations were stronger and more consistently significant in ARIC, likely reflecting its larger sample size, longer follow-up, and greater number of events. In MESA, estimates trended in the same direction but were less precise due to shorter LDL-C observation windows and fewer events. The cohorts also reflect different eras of lipid-lowering therapy: ARIC participants were followed primarily during the late 1980s-1990s, after the release of the National Cholesterol Education Program’s Adult Treatment Panel (ATP) I and II guidelines [40,41], when statin use was still emerging, whereas MESA participants were enrolled after 2000, in the ATP III era [42], when statins were widely recommended [43]. This is reflected in cohorts’ characteristic, ARIC had lower lipid-lowering medication use, higher mean LDL-C and lower TTR levels, whereas MESA had greater lipid-lowering therapy use, lower LDL-C, and higher TTR. Despite these differences, Fine-Gray competing risk models yielded broadly consistent associations, supporting the predictive value of LDL-C TTR across populations differing in baseline characteristics, treatment eras, and lipid-lowering patterns.

Differences in LDL-C measurement frequency and follow-up between cohorts deserve mention. In ARIC, 83.2 % of participants had four measurements over 8.6 years on average (mean interval 3.1 years), whereas in MESA, 92.3 % had four measurements over 4.8 years (mean interval 1.6 years). Although frequent measurements would provide finer resolution of short-term variability, LDL-C is not routinely monitored at short intervals as are blood pressure or glucose, for which dynamic or continuous monitoring is feasible in clinical practice [[44], [45], [46]]. Even in randomized clinical trials, LDL-C is typically assessed every 3–6 months or annually [47,48], and most guidelines recommend annual or multi-yearly assessments [6,49]. In large community-based cohort studies, frequent LDL-C measurements are even rarer. Our data therefore reflect real-world practice, yet TTR derived from these relatively sparse data still captured long-term exposure patterns and showed robust associations with cardiovascular outcomes. These considerations also underscore an important limitation, namely that LDL-C was measured at relatively long intervals, which we further acknowledge below.

Our findings suggest that LDL-C TTR, which integrates both the magnitude and duration of LDL-C control, could serve as a practical metric for monitoring long-term cholesterol control. Calculable from as few as three to four LDL-C measurements, compatible with routine clinical follow-up, TTR can be incorporated into electronic health record dashboards to help clinicians track patients’ LDL-C control over time. By identifying individuals with persistently borderline or elevated LDL-C, TTR provides actionable opportunities for targeted interventions and improved risk stratification. These applications align with contemporary guideline-directed cholesterol management and precision cardiovascular risk reduction strategies, supporting individualized preventive care.

### 4.1 Limitations

Our study has several limitations. First, LDL-C was measured at relatively long intervals (mean 3.1 years in ARIC and 1.6 years in MESA), which may underestimate short-term fluctuations. However, such measurement frequencies reflect real-world clinical and epidemiological practice, and the consistency of associations across cohorts suggests that LDL-C TTR remains robust despite less frequent sampling. Second, LDL-C was calculated rather than directly assayed, and participants with TC >400 mg/dL were excluded, which may lead to some loss of information and potential systematic error. Nonetheless, the use of standardized Friedewald-based methods and exclusion of extreme values likely minimized bias. Third, pooling ARIC and MESA increased power and diversity but may also have introduced heterogeneity given differences in baseline characteristics, follow-up duration, and event adjudication. Nonetheless, further analyses stratified by cohort confirmed that the results were directionally consistent, even after adjustment for baseline factors. Fourth, both cohorts were US-based and analyses used US guidelines, which may limit generalizability to other population. Finally, the observational design precludes causal inference, interventional trials are needed to determine whether sustained LDL-C TTR directly reduces CVD events.

## 5 Conclusions

LDL-C TTR is independently associated with a decreased risk of MACE over time, beyond what current LDL-C measures and indices provide. TTR may serve as a valuable metric for long-term LDL-C control and patient management. Future research should focus on validating LDL-C TTR in other populations and exploring its potential as a target for intervention.

## Ethics approval

This study involved secondary data analysis, it was granted a waiver of ethical approval by the Ethical Review Board of the First Affiliated Hospital of Xi’an Jiaotong University.

## Funding

This work was supported by the National Natural Science Foundation of China, 82322060, 81803264.

## CRediT authorship contribution statement

**Yezhou Liu:** Writing – original draft, Formal analysis, Conceptualization. **Zhe Lv:** Writing – original draft, Formal analysis. **Hong Yan:** Writing – review & editing. **Yamei Liu:** Writing – review & editing. **Jiaheng Zhang:** Writing – review & editing. **Wenming Bian:** Writing – review & editing. **Yetong Liu:** Writing – review & editing. **Zhaojie Song:** Writing – review & editing. **Peng Han:** Writing – review & editing. **Tao Chen:** Writing – review & editing, Supervision. **Chao Li:** Writing – review & editing, Supervision, Funding acquisition, Conceptualization.

## Declaration of competing interest

The authors declare the following financial interests/personal relationships which may be considered as potential competing interests: Chao Li reports article publishing charges was provided by National Natural Science Foundation of China. If there are other authors, they declare that they have no known competing financial interests or personal relationships that could have appeared to influence the work reported in this paper.

## Appendix Supplementary materials

Image, application 1
