# Supplementary material for: Time in LDL cholesterol target range and major adverse cardiovascular events risk: A pooled analysis of two cohorts
Source: Am J Prev Cardiol. 2025 Sep 10;24:101290. doi: 10.1016/j.ajpc.2025.101290 (PMC12746287; doi:10.1016/j.ajpc.2025.101290)
Supplement: Supplementary file 1 [file mmc1.docx]

**Supplementary Materials**

| **Supplementary Table 1 Characteristics of participants from 2 cohorts** | | | |
| --- | --- | --- | --- |
| **Characteristic** | **Overall** | **ARIC** | **MESA** |
| N | 16,310 | 10,835 (66.4) | 5,475 (33.6) |
| Gender |  |  |  |
| Female | 9,127 (56.0) | 6,205 (57.3) | 2,922 (53.4) |
| Male | 7,183 (44.0) | 4,630 (42.7) | 2,553 (46.6) |
| Age, years | 56.4 ± 8.2 | 53.9 ± 5.7 | 61.5 ± 9.9 |
| White | 10,701 (65.6) | 8,485 (78.3) | 2,216 (40.5) |
| Educational level |  |  |  |
| Below high school | 3,889 (23.9) | 2,054 (19.0) | 1,835 (33.6) |
| High school graduate or GED | 5,853 (36.0) | 4,577 (42.3) | 1,276 (23.4) |
| College or above | 6,538 (40.2) | 4,186 (38.7) | 2,352 (43.1) |
| Smoking |  |  |  |
| Never | 7,666 (47.1) | 4,888 (45.2) | 2,778 (50.9) |
| Former | 5,564 (34.2) | 3,528 (32.6) | 2,036 (37.3) |
| Current | 3,059 (18.8) | 2,410 (22.3) | 649 (11.9) |
| Drinking |  |  |  |
| Never | 3,734 (23.0) | 2,651 (24.6) | 1,083 (19.9) |
| Former | 3,025 (18.6) | 1,791 (16.6) | 1,234 (22.7) |
| Current | 9,479 (58.4) | 6,354 (58.9) | 3,125 (57.4) |
| BMI, kg/m^2^ | 27.8 ± 5.3 | 27.5 ± 5.2 | 28.3 ± 5.4 |
| MVPA, MET-min/wk | 955.6 ± 1,603.3 | 620.5 ± 767.0 | 1,619.6 ± 2,415.9 |
| Cholesterol-lowering Medicines Use | 1,139 (7.0) | 264 (2.5) | 875 (16.0) |
| Hypertension | 5,578 (34.3) | 3,230 (29.9) | 2,348 (42.9) |
| Diabetes | 1,533 (9.4) | 934 (8.7) | 599 (11.0) |
| Number of LDL-C measurements |  |  |  |
| 3 | 2,239 (13.7) | 1,817 (16.8) | 422 (7.7) |
| 4 | 14,071 (86.3) | 9,018 (83.2) | 5,053 (92.3) |
| Duration of LDL-C measurements, years | 7.3 ± 2.0 | 8.6 ± 1.0 | 4.8 ± 0.4 |
| Mean interval between LDL-C measurements, years | 2.6 ± 0.7 | 3.1 ± 0.3 | 1.6 ± 0.2 |
| Mean LDL-C, mg/dL | 124.4 ± 30.5 | 129.9 ± 30.8 | 113.4 ± 26.5 |
| LDL-C SD, mg/dL | 18.0 ± 11.6 | 18.5 ± 11.8 | 17.1 ± 11.2 |
| LDL-C CV | 0.1 ± 0.1 | 0.1 ± 0.1 | 0.2 ± 0.1 |
| LDL-C ARV, mg/dL | 20.5 ± 13.8 | 21.1 ± 14.1 | 19.3 ± 13.2 |
| LDL-C TTR, % | 80.6 ± 14.3 | 78.0 ± 14.4 | 85.8 ± 12.5 |
| Data are shown as n (%) or Mean ± SD. There were 30 missing values for educational level, 21 for smoking, 72 for drinking, 8 for BMI, 18 for MVPA sports, 81 for cholesterol-lowering use, 44 for hypertension, and 65 for diabetes. Abbreviations: ARIC: Atherosclerosis Risk in Communities study; MESA: Multi-Ethnic Study of Atherosclerosis; GED, general educational development; BMI: body mass index; MVPA: moderate and vigorous physical activity; LDL-C: low density lipoprotein cholesterol; SD: Standard deviation; CV: coefficient of variation; ARV, average real variability; TTR: time in target range. SI conversion factor: To convert cholesterol levels to millimoles per liter, multiply by 0.0259 | | | |


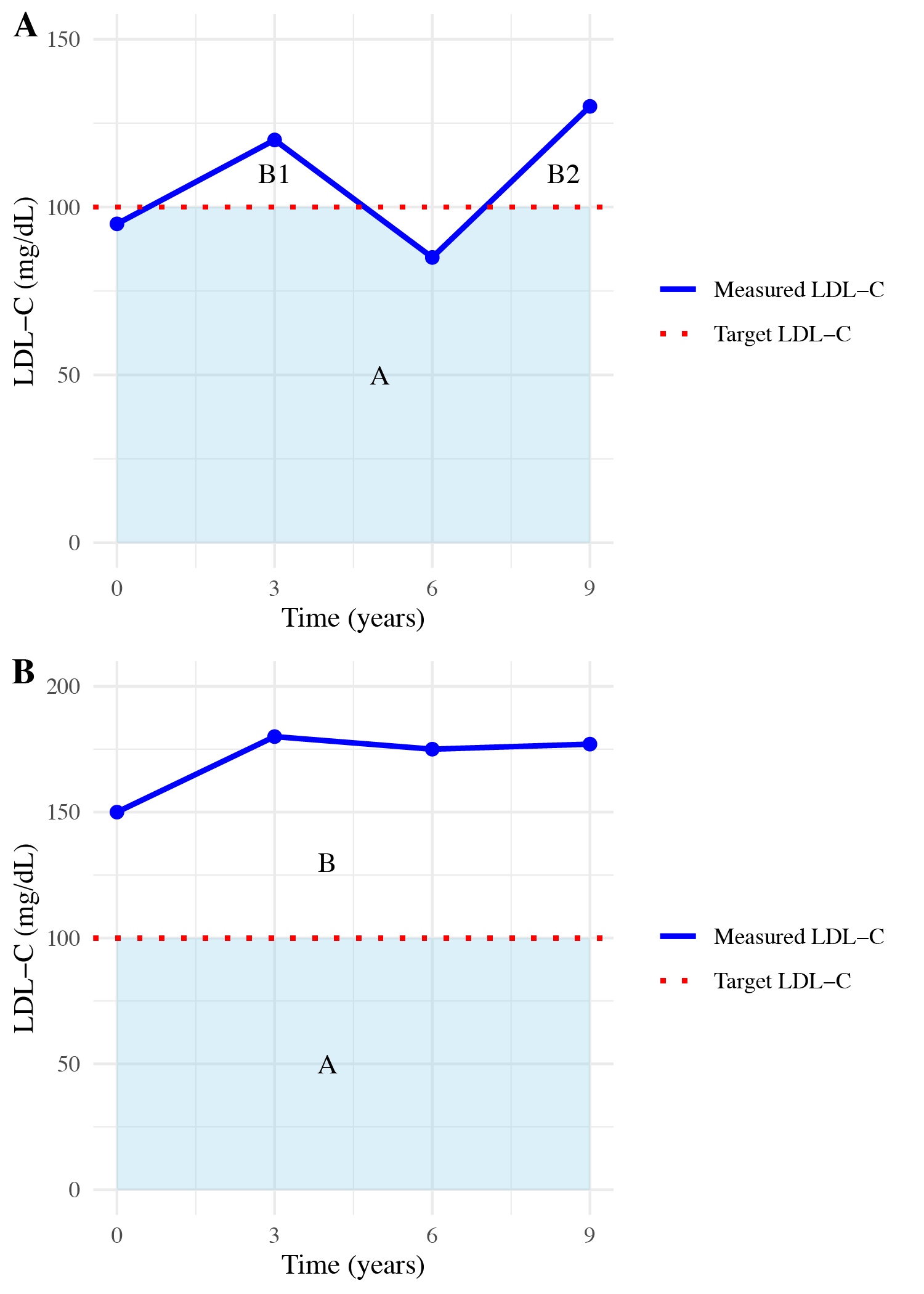


**Supplementary Figure 1** **Example calculation of LDL-C TTR**

Plot A: TTR = A / (A + B1 + B2) * 100; Plot B: TTR = A / (A + B) * 100


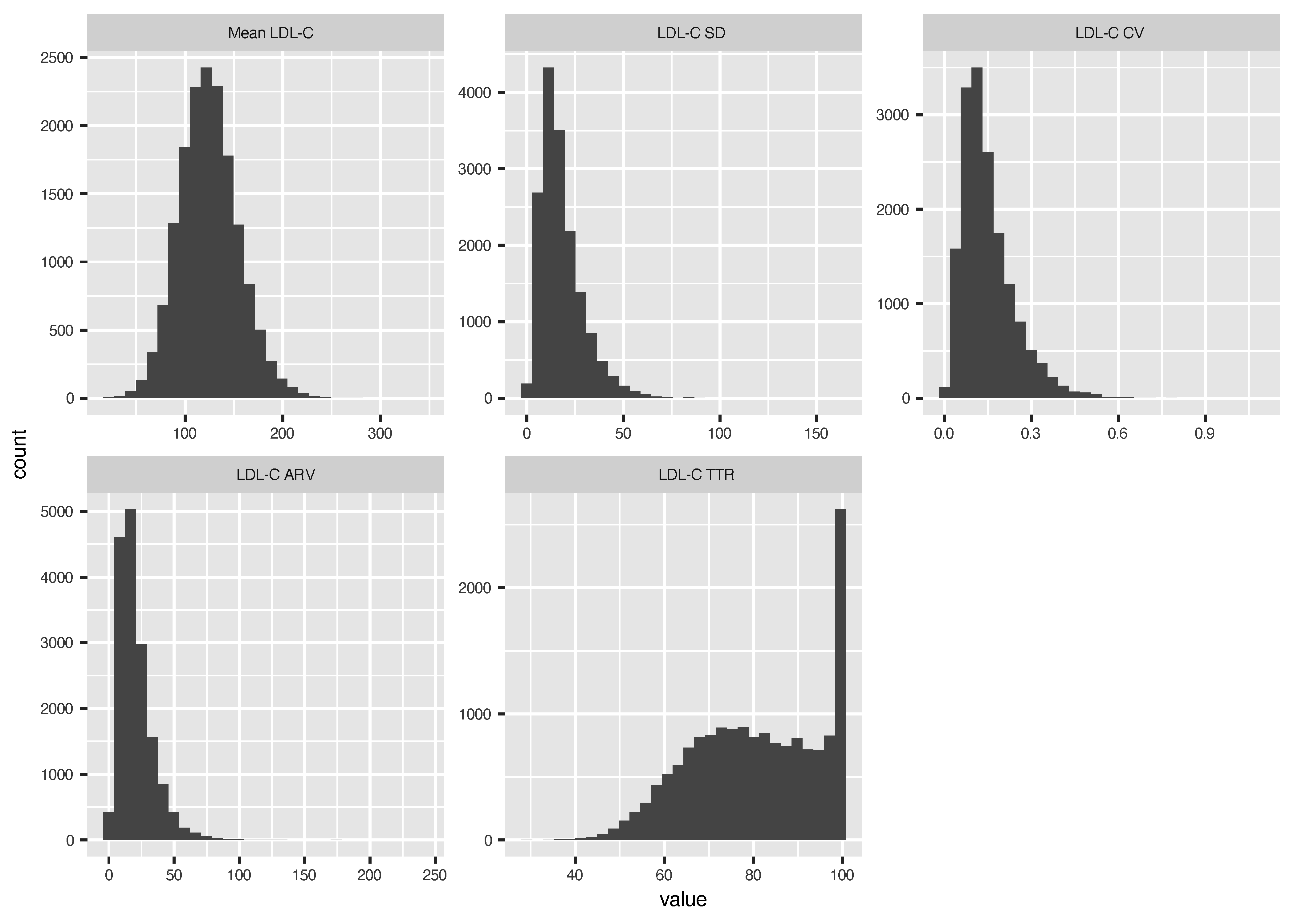


**Supplementary Figure 2 distribution of LDL-C Indices**


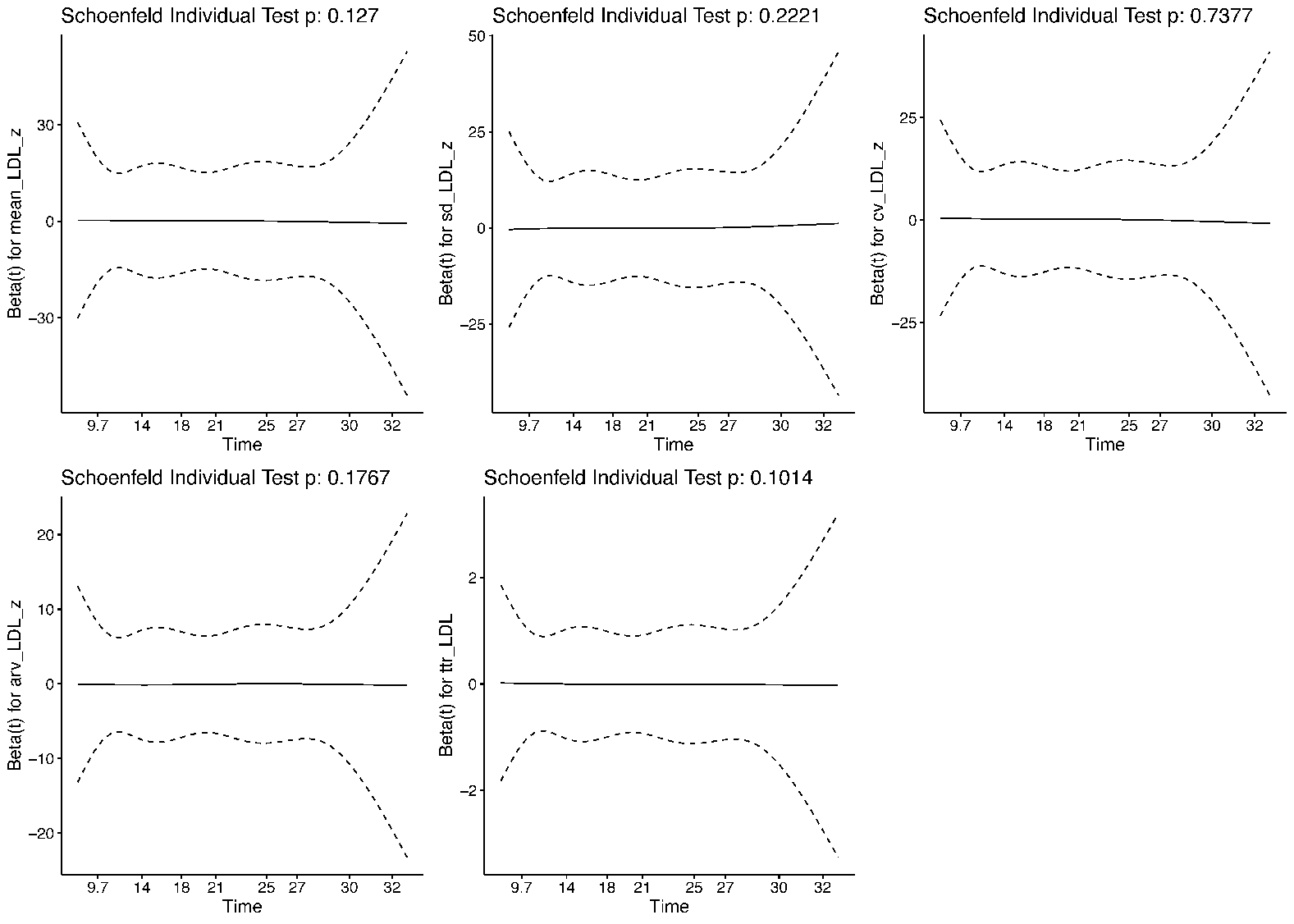


**Supplementary Figure 3 Schoenfeld Residual Plots for Each LDL-C Indices**


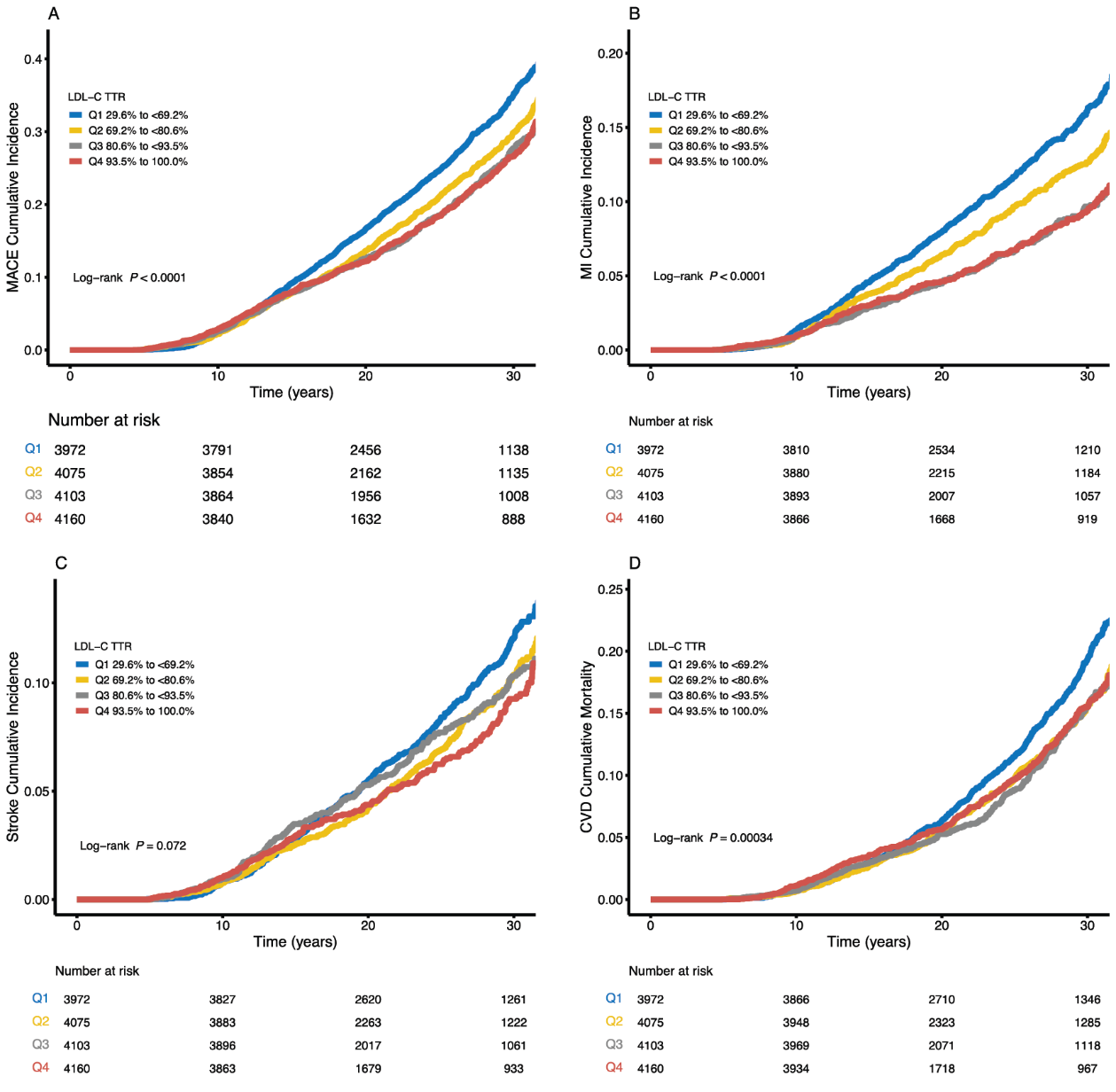


**Supplementary Figure 4 Incidence Curve of Cardiovascular Outcomes by LDL-C TTR** **Quartiles**

A: MACE; B: MI; C: Stroke; D: CVD Death

MACE: major adverse cardiovascular event; MI: myocardial infarction; LDL-C: low density lipoprotein cholesterol; and TTR: time in target range.

MACE was defined as the first occurrence of MI, stroke, and cardiovascular death.

| **Supplementary Table 2 Associations of LDL-C TTR and cardiovascular outcomes by cohort, HR (95%CI)** | | | | | | | |
| --- | --- | --- | --- | --- | --- | --- | --- |
|  |  |  | **Quarters of LDL-C TTR** | | | |  |
|  | **N** | **N of Event** | **Q1** | **Q2** | **Q3** | **Q4** | ***P* for trend** |
| **ARIC** |  |  |  |  |  |  |  |
| MACE | 10,620 | 2,799 | 1 (ref.) | 0.86 (0.78 to 0.95) | 0.77 (0.70 to 0.86) | 0.78 (0.70 to 0.87) | <0.001 |
| MI | 10,620 | 1,085 | 1 (ref.) | 0.80 (0.69 to 0.93) | 0.60 (0.50 to 0.70) | 0.55 (0.46 to 0.66) | <0.001 |
| Stroke | 10,620 | 898 | 1 (ref.) | 0.86 (0.72 to 1.03) | 0.86 (0.72 to 1.02) | 0.78 (0.65 to 0.94) | 0.012 |
| CVD Death | 10,620 | 1,492 | 1 (ref.) | 0.89 (0.77 to 1.02) | 0.83 (0.72 to 0.95) | 0.93 (0.80 to 1.07) | 0.14 |
| **MESA** |  |  |  |  |  |  |  |
| MACE | 5,426 | 457 | 1 (ref.) | 0.87 (0.67 to 1.14) | 0.81 (0.62 to 1.06) | 0.77 (0.59 to 1.01) | 0.055 |
| MI | 5,426 | 180 | 1 (ref.) | 0.76 (0.50 to 1.16) | 0.78 (0.51 to 1.18) | 0.69 (0.45 to 1.06) | 0.12 |
| Stroke | 5,426 | 177 | 1 (ref.) | 0.80 (0.52 to 1.23) | 0.88 (0.58 to 1.33) | 0.71 (0.46 to 1.10) | 0.18 |
| CVD Death | 5,426 | 178 | 1 (ref.) | 1.08 (0.69 to 1.69) | 0.83 (0.52 to 1.33) | 1.08 (0.70 to 1.69) | 0.93 |
| Abbreviation: HR indicates hazard ratio; MACE: major adverse cardiovascular event; CVD: cardiovascular disease; MI: myocardial infarction; LDL-C, low density lipoprotein cholesterol; and TTR, Time in target range. MACE was defined as the first occurrence of MI, stroke, and cardiovascular death.  Cox models were deployed. Adjusted for age, gender, race, education level, smoking status, drinking status, body mass index, moderate-vigorous physical activity, use of cholesterol-lowering medication, prevalent hypertension, and prevalent diabetes. | | | | | | | |

| **Supplementary Table 2a Associations of LDL-C TTR and cardiovascular outcomes pooled and stratified by cohort, sHR (95% CI)** | | | | | | | |
| --- | --- | --- | --- | --- | --- | --- | --- |
|  |  |  | **Quartiles of LDL-C TTR** | | | |  |
|  | **N of event** | **N of competing event** | **Q1** | **Q2** | **Q3** | **Q4** | ***P* for trend** |
| **ALL** |  |  |  |  |  |  |  |
| MACE | 3256 | 3508 | 1 (ref.) | 0.83 (0.76 to 0.91) | 0.77 (0.70 to 0.85) | 0.71 (0.64 to 0.78) | <0.001 |
| MI | 1265 | 5137 | 1 (ref.) | 0.80 (0.69 to 0.92) | 0.60 (0.51 to 0.70) | 0.58 (0.49 to 0.68) | <0.001 |
| Stroke | 1075 | 5234 | 1 (ref.) | 0.88 (0.75 to 1.04) | 0.93 (0.79 to 1.10) | 0.75 (0.63 to 0.90) | 0.005 |
| CVD death | 1670 | 4222 | 1 (ref.) | 0.79 (0.70 to 0.90) | 0.80 (0.70 to 0.91) | 0.77 (0.67 to 0.88) | <0.001 |
| **ARIC** |  |  |  |  |  |  |  |
| MACE | 2799 | 3012 | 1 (ref.) | 0.85 (0.77 to 0.94) | 0.79 (0.71 to 0.88) | 0.75 (0.67 to 0.84) | <0.001 |
| MI | 1085 | 4466 | 1 (ref.) | 0.80 (0.69 to 0.93) | 0.58 (0.49 to 0.69) | 0.57 (0.48 to 0.69) | <0.001 |
| Stroke | 898 | 4561 | 1 (ref.) | 0.91 (0.77 to 1.08) | 0.92 (0.77 to 1.11) | 0.76 (0.62 to 0.93) | 0.015 |
| CVD death | 1492 | 3661 | 1 (ref.) | 0.81 (0.71 to 0.93) | 0.84 (0.73 to 0.97) | 0.83 (0.71 to 0.97) | 0.013 |
| **MESA** |  |  |  |  |  |  |  |
| MACE | 457 | 496 | 1 (ref.) | 0.71 (0.51 to 0.99) | 0.71 (0.52 to 0.97) | 0.59 (0.44 to 0.80) | 0.002 |
| MI | 180 | 671 | 1 (ref.) | 0.71 (0.43 to 1.17) | 0.60 (0.37 to 0.96) | 0.51 (0.33 to 0.81) | 0.005 |
| Stroke | 177 | 673 | 1 (ref.) | 0.67 (0.39 to 1.16) | 0.88 (0.54 to 1.43) | 0.66 (0.40 to 1.07) | 0.22 |
| CVD death | 178 | 561 | 1 (ref.) | 0.83 (0.46 to 1.49) | 0.77 (0.44 to 1.34) | 0.76 (0.45 to 1.31) | 0.39 |
| Abbreviations: sHR indicates subdistribution hazard ratio; MACE: major adverse cardiovascular event; CVD: cardiovascular disease; MI: myocardial infarction; LDL-C, low-density lipoprotein cholesterol; and TTR, time in target range. MACE was defined as the first occurrence of MI, stroke, and cardiovascular death. Fine–Gray competing risk models were used. For MACE and CVD death, non-CVD death was treated as a competing event; for MI and stroke, all-cause death was treated as a competing event. Adjusted for age, gender, race, education level, smoking status, drinking status, body mass index, moderate-to-vigorous physical activity, use of cholesterol-lowering medication, prevalent hypertension, and prevalent diabetes. | | | | | | | |

| **Supplementary Table 3 Association Between LDL-C Indices With Cardiovascular Outcomes** | | | | | | | | | | | | | |
| --- | --- | --- | --- | --- | --- | --- | --- | --- | --- | --- | --- | --- | --- |
|  |  |  |  | **LDL-C TTR** | | **Mean LDL-C** | | **LDL-C SD** | | **LDL-C CV** | | **LDL-C ARV** | |
|  |  | **N** | **N of Event** | **HR (95% CI)** | ***P* value** | **HR (95% CI)** | ***P* value** | **HR (95% CI)** | ***P* value** | **HR (95% CI)** | ***P* value** | **HR (95% CI)** | ***P* value** |
| MACE | Unadjusted | 16,280 | 3,306 | 0.87 (0.84 to 0.90) | <0.001 | 1.15 (1.11 to 1.19) | <0.001 | 1.10 (1.07 to 1.14) | <0.001 | 1.06 (1.02 to 1.10) | 0.002 | 1.07 (1.04 to 1.11) | <0.001 |
|  | Model 1 | 16,280 | 3,306 | 0.90 (0.87 to 0.93) | <0.001 | 1.11 (1.07 to 1.15) | <0.001 | 1.10 (1.06 to 1.13) | <0.001 | 1.07 (1.03 to 1.11) | <0.001 | 1.08 (1.05 to 1.12) | <0.001 |
|  | Model 2 | 16,204 | 3,291 | 0.90 (0.87 to 0.93) | <0.001 | 1.10 (1.06 to 1.14) | <0.001 | 1.09 (1.05 to 1.13) | <0.001 | 1.06 (1.03 to 1.10) | <0.001 | 1.08 (1.05 to 1.12) | <0.001 |
|  | Model 3 | 16,046 | 3,256 | 0.89 (0.86 to 0.92) | <0.001 | 1.12 (1.08 to 1.16) | <0.001 | 1.07 (1.03 to 1.10) | <0.001 | 1.03 (1.00 to 1.07) | 0.091 | 1.06 (1.03 to 1.10) | <0.001 |
|  |  |  |  |  |  |  |  |  |  |  |  |  |  |
| MI | Unadjusted | 16,280 | 1,288 | 0.77 (0.73 to 0.82) | <0.001 | 1.28 (1.21 to 1.35) | <0.001 | 1.17 (1.12 to 1.23) | <0.001 | 1.11 (1.05 to 1.17) | <0.001 | 1.15 (1.09 to 1.20) | <0.001 |
|  | Model 1 | 16,280 | 1,288 | 0.79 (0.74 to 0.83) | <0.001 | 1.26 (1.19 to 1.33) | <0.001 | 1.18 (1.13 to 1.24) | <0.001 | 1.12 (1.06 to 1.19) | <0.001 | 1.17 (1.11 to 1.22) | <0.001 |
|  | Model 2 | 16,204 | 1,280 | 0.79 (0.75 to 0.84) | <0.001 | 1.24 (1.18 to 1.32) | <0.001 | 1.18 (1.12 to 1.24) | <0.001 | 1.12 (1.06 to 1.19) | <0.001 | 1.17 (1.11 to 1.22) | <0.001 |
|  | Model 3 | 16,046 | 1,265 | 0.78 (0.74 to 0.83) | <0.001 | 1.27 (1.20 to 1.34) | <0.001 | 1.16 (1.10 to 1.22) | <0.001 | 1.09 (1.03 to 1.15) | 0.002 | 1.15 (1.10 to 1.21) | <0.001 |
|  |  |  |  |  |  |  |  |  |  |  |  |  |  |
| Stroke | Unadjusted | 16,280 | 1,090 | 0.90 (0.84 to 0.95) | <0.001 | 1.12 (1.05 to 1.19) | <0.001 | 1.04 (0.98 to 1.10) | 0.26 | 0.98 (0.92 to 1.05) | 0.57 | 1.00 (0.94 to 1.06) | 0.88 |
|  | Model 1 | 16,280 | 1,090 | 0.93 (0.87 to 0.99) | 0.017 | 1.08 (1.02 to 1.14) | 0.014 | 1.03 (0.97 to 1.09) | 0.41 | 0.99 (0.92 to 1.05) | 0.65 | 1.00 (0.94 to 1.06) | 0.96 |
|  | Model 2 | 16,204 | 1,085 | 0.93 (0.87 to 0.99) | 0.017 | 1.08 (1.01 to 1.14) | 0.015 | 1.02 (0.96 to 1.08) | 0.52 | 0.98 (0.92 to 1.05) | 0.56 | 1.00 (0.94 to 1.06) | 0.95 |
|  | Model 3 | 16,046 | 1,075 | 0.91 (0.86 to 0.97) | 0.004 | 1.10 (1.03 to 1.17) | 0.002 | 1.00 (0.94 to 1.06) | 0.99 | 0.95 (0.89 to 1.01) | 0.13 | 0.98 (0.92 to 1.05) | 0.61 |
|  |  |  |  |  |  |  |  |  |  |  |  |  |  |
| CVD Death | Unadjusted | 16,280 | 1,694 | 0.91 (0.86 to 0.95) | <0.001 | 1.09 (1.04 to 1.14) | <0.001 | 1.12 (1.08 to 1.17) | <0.001 | 1.11 (1.06 to 1.16) | <0.001 | 1.08 (1.03 to 1.13) | 0.001 |
|  | Model 1 | 16,280 | 1,694 | 0.96 (0.92 to 1.01) | 0.12 | 1.02 (0.98 to 1.07) | 0.35 | 1.11 (1.07 to 1.17) | <0.001 | 1.12 (1.07 to 1.18) | <0.001 | 1.08 (1.04 to 1.13) | <0.001 |
|  | Model 2 | 16,204 | 1,689 | 0.97 (0.92 to 1.01) | 0.16 | 1.02 (0.97 to 1.07) | 0.45 | 1.10 (1.05 to 1.15) | <0.001 | 1.11 (1.06 to 1.17) | <0.001 | 1.08 (1.03 to 1.13) | 0.001 |
|  | Model 3 | 16,046 | 1,670 | 0.95 (0.90 to 1.00) | 0.043 | 1.04 (0.99 to 1.09) | 0.14 | 1.08 (1.03 to 1.13) | 0.001 | 1.08 (1.03 to 1.13) | 0.003 | 1.06 (1.01 to 1.11) | 0.016 |
| HR per 1-SD increase in LDL-C TTR, mean LDL-C, LDL-C SD, and LDL-C ARV.  MACE was defined as the first occurrence of MI, stroke, and cardiovascular death. Abbreviation:  HR: hazard ratio; CI: confidence interval; MACE: major adverse cardiovascular event; MI: myocardial infarction; LDL-C: low density lipoprotein cholesterol; TTR: time in target range; SD: standard deviation; CV: coefficient of variation; and ARV, average real variability. Cox models with cohort strata were deployed. Model 1: adjusted for age, gender, race, and education level;  Model 2: adjusted for model 1 + smoking status, drinking status, body mass index, and moderate-vigorous physical activity;  Model 3: adjusted for model 2 + use of cholesterol- lowering drugs, prevalent hypertension, and prevalent diabetes. | | | | | | | | | | | | | |

| **Supplementary Table 4 Cross-adjusted Association of LDL-C TTR and LDL-C Indices and Cardiovascular Outcomes** | | | | | | | | | | | |
| --- | --- | --- | --- | --- | --- | --- | --- | --- | --- | --- | --- |
|  | **MACE** | |  | **MI** | |  | **Stroke** | |  | **CVD Death** | |
|  | **HR (95% CI)** | ***P* value** |  | **HR (95% CI)** | ***P* value** |  | **HR (95% CI)** | ***P* value** |  | **HR (95% CI)** | ***P* value** |
| **Without cross-adjustment** |  |  |  |  |  |  |  |  |  |  |  |
| LDL-C TTR | 0.89 (0.86 to 0.92) | <0.001 |  | 0.78 (0.74 to 0.83) | <0.001 |  | 0.91 (0.86 to 0.97) | 0.004 |  | 0.95 (0.90 to 1.00) | 0.043 |
| **Cross-adjusted with Mean** |  |  |  |  |  |  |  |  |  |  |  |
| LDL-C TTR | 0.90 (0.79 to 1.04) | 0.15 |  | 0.81 (0.65 to 1.00) | 0.053 |  | 1.01 (0.80 to 1.27) | 0.94 |  | 0.80 (0.67 to 0.97) | 0.024 |
| Mean LDL-C | 1.02 (0.89 to 1.17) | 0.78 |  | 1.03 (0.83 to 1.28) | 0.76 |  | 1.11 (0.88 to 1.39) | 0.38 |  | 0.84 (0.70 to 1.02) | 0.074 |
| **Cross-adjusted with SD** |  |  |  |  |  |  |  |  |  |  |  |
| LDL-C TTR | 0.90 (0.86 to 0.93) | <0.001 |  | 0.80 (0.76 to 0.85) | <0.001 |  | 0.90 (0.85 to 0.96) | 0.002 |  | 0.97 (0.92 to 1.03) | 0.31 |
| LDL-C SD | 1.03 (1.00 to 1.07) | 0.077 |  | 1.08 (1.03 to 1.14) | 0.003 |  | 0.97 (0.91 to 1.03) | 0.33 |  | 1.07 (1.02 to 1.12) | 0.008 |
| **Cross-adjusted with CV** |  |  |  |  |  |  |  |  |  |  |  |
| LDL-C TTR | 0.89 (0.86 to 0.92) | <0.001 |  | 0.78 (0.73 to 0.82) | <0.001 |  | 0.91 (0.86 to 0.97) | 0.005 |  | 0.95 (0.90 to 1.00) | 0.034 |
| LDL-C CV | 1.04 (1.00 to 1.07) | 0.054 |  | 1.10 (1.04 to 1.16) | <0.001 |  | 0.95 (0.89 to 1.02) | 0.16 |  | 1.08 (1.03 to 1.13) | 0.002 |
| **Cross-adjusted with ARV** |  |  |  |  |  |  |  |  |  |  |  |
| LDL-C TTR | 0.90 (0.86 to 0.93) | <0.001 |  | 0.80 (0.75 to 0.85) | <0.001 |  | 0.90 (0.84 to 0.96) | 0.001 |  | 0.96 (0.92 to 1.02) | 0.17 |
| LDL-C ARV | 1.03 (0.99 to 1.07) | 0.1 |  | 1.08 (1.03 to 1.14) | 0.003 |  | 0.95 (0.89 to 1.02) | 0.15 |  | 1.05 (1.00 to 1.10) | 0.063 |
| **Cross-adjusted with Mean and SD** |  |  |  |  |  |  |  |  |  |  |  |
| LDL-C TTR | 0.92 (0.80 to 1.06) | 0.24 |  | 0.84 (0.68 to 1.04) | 0.11 |  | 0.99 (0.78 to 1.25) | 0.95 |  | 0.84 (0.70 to 1.02) | 0.073 |
| Mean LDL-C | 1.03 (0.90 to 1.17) | 0.7 |  | 1.05 (0.85 to 1.29) | 0.67 |  | 1.10 (0.87 to 1.39) | 0.42 |  | 0.86 (0.71 to 1.04) | 0.12 |
| LDL-C SD | 1.03 (1.00 to 1.07) | 0.073 |  | 1.08 (1.03 to 1.14) | 0.003 |  | 0.97 (0.91 to 1.04) | 0.36 |  | 1.07 (1.01 to 1.12) | 0.011 |
| HR per 1-SD increase in LDL-C TTR, mean LDL-C, LDL-C SD, LDL-C CV, and LDL-C ARV. Abbreviation:  HR: hazard ratio; CI: confidence interval; MACE: major adverse cardiovascular event; MI: myocardial infarction; LDL-C: low density lipoprotein cholesterol; TTR: time in target range; SD: standard deviation; CV: coefficient of variation; and ARV, average real variability. Cox models with cohort strata were deployed. Adjusted for age, gender, race, education level, smoking status, drinking status, body mass index, moderate-vigorous physical activity use of lowering-cholesterol drugs, prevalent hypertension, and prevalent diabetes. | | | | | | | | | | | |

| **Supplementary Table 5 Prognostic Value of LDL-C Indices Compared With Traditional Risk Factors in MACE** | | | |
| --- | --- | --- | --- |
| **Model** | **AIC** | **C-statistics (95% CI)** | **C-statistics Difference (95% CI)** |
| Base model* | 57167.81 | 0.715 (0.706 to 0.725) |  |
| Base model + LDL-C TTR | 57127.97 | 0.718 (0.709 to 0.727) | 0.0026 (-0.0103 to 0.0155) |
| Base model + Mean LDL-C | 57130.00 | 0.718 (0.709 to 0.727) | 0.0024 (-0.0105 to 0.0153) |
| Base model + LDL-C SD | 57155.35 | 0.716 (0.707 to 0.725) | 0.0009 (-0.0121 to 0.0138) |
| Base model + LDL-C CV | 57167.03 | 0.716 (0.707 to 0.725) | 0.0002 (-0.0127 to 0.0131) |
| Base model + LDL-C ARV | 57157.46 | 0.716 (0.707 to 0.725) | 0.0007 (-0.0123 to 0.0136) |
| Abbreviations: LDL-C: low density lipoprotein cholesterol; SD: Standard deviation; CV: coefficient of variation; ARV, average real variability; TTR: time in target range. Cox models with cohort strata were deployed. *Adjusted for age, gender, race, education level, smoking status, drinking status, body mass index, moderate-vigorous physical activity, use of cholesterol-lowering medicines, prevalent hypertension, and prevalent diabetes. | | | |
